# Diagnostic Dilemmas in Giant Cell Arteritis: Overcoming Anchoring Bias

**DOI:** 10.1155/crrh/6632374

**Published:** 2025-07-21

**Authors:** Crystal Stewart, Rana H. Asif, Tahani Dakkak, Hardeep Singh, Muhammad Ali Javaid, Nikesh Patel

**Affiliations:** ^1^Graduate Medical Education Research Department, Northeast Georgia Medical Center, Gainesville, Georgia, USA; ^2^Internal Medicine GME Program, Northeast Georgia Medical Center, Gainesville, Georgia 30501, USA

**Keywords:** C-reactive protein, fever, fever of unknown origin, giant cell arteritis, temporal artery biopsy

## Abstract

Giant cell arteritis (GCA), also known as temporal arteritis, is the most common systemic vasculitis in individuals over 50 and presents diagnostic challenges due to its nonspecific symptoms such as fever, headache, and fatigue. This case report describes the details of a male patient in his 70s who presented with recurrent intermittent fevers of unknown origin and was ultimately diagnosed with GCA after an extensive workup. His initial CT scans and lab tests were unremarkable. However, after a rheumatological workup displayed elevated erythrocyte sedimentation rate (ESR) and C-reactive protein (CRP) levels, along with new symptoms of ataxia and headaches, a temporal artery biopsy (TAB) was performed and confirmed the patient had GCA. This case underscores the difficulty in diagnosing GCA primarily due to physician anchoring bias, particularly when typical symptoms are not present. The case also showcases the need for increased awareness and prompt evaluation of potential GCA symptoms to prevent severe complications. Public education as well as improved hospital protocols can lead to earlier detection and treatment of GCA, reducing the risk of morbidity.

## 1. Introduction

Giant cell arteritis (GCA), also referred to as temporal arteritis, is the most common form of systemic vasculitis, with an incidence of between 18 and 29 cases per 100,000 persons over 50 years of age. GCA causes chronic inflammation of the blood vessels that predominantly affects large and medium-sized arteries, primarily the cranial branches of the carotid arteries [1, 2]. GCA affects women with a lifetime risk of 1% compared to 0.5% in men [3]. The most commonly associated symptoms of GCA can vary widely, including fever, new-onset headaches, jaw claudication, visual disturbances, scalp tenderness, arthralgias, and joint stiffness in addition to general symptoms like fatigue, malaise, and weight loss [4, 5]. There has been limited evidence of GCA presentation in the absence of the aforementioned commonly associated manifestations. It is often associated with severe complications such as irreversible vision loss due to anterior ischemic optic neuropathy and stroke making it one of the few rheumatologic emergencies [3, 6]. These patients have been shown to be at a 40% increased risk for strokes compared to the general population [6].

Aside from stroke and permanent vision loss, some rare complications of GCA include thoracic aortic aneurysms and dissections, carotid and vertebral artery dissection, ischemic heart disease, and peripheral vascular disease [6–8]. There also have been reports of rare ischemic complications including scalp and tongue necrosis and sixth nerve palsy [9]. The most common comorbidity of GCA is polymyalgia rheumatica, a rheumatic disorder characterized by pain and stiffness around the neck, shoulder, and hip area [10]. Approximately 40%–60% of patients with GCA also have polymyalgia rheumatica [11, 12]. Current treatment guidelines for GCA recommend high-dose glucocorticoids to reduce arterial inflammation and prevent further complication. Treatment plans also involve managing associated conditions, regular monitoring, and possible surgery for blood vessel complications [5, 13]. This case report presents an ambiguous case, highlighting the complexities involved in diagnosing GCA.

## 2. Case Presentation

A male patient in his 70s presented to the emergency department with recurring fevers for approximately 4 weeks, with a peak temperature of 103° F. The patient was a relatively poor historian. He reported having intermittent fevers, the last three of which were more persistent. He was taking Tylenol 1000 mg every 6 h along with ibuprofen as needed to break his fever. He was evaluated by his PCP, and in the emergency room (ER) during this time, his workup remained unremarkable although erythrocyte sedimentation rate (ESR) and C-reactive protein (CRP) were not checked. He reported completing a course of Augmentin as well, but symptoms persisted. The patient had a history of hypertension, hyperlipidemia, depression, and low vitamin D levels. He denied any recent travels and any other associated symptoms with the fevers. While in the emergency department, the patient was hemodynamically stable and afebrile and reported taking acetaminophen prior to arrival.

On physical exam, patient was alert and oriented with no focal neurological deficits, normal S1 and S2, lungs were clear to auscultation, no abdominal tenderness with normal bowel sounds, no joint tenderness, swelling or stiffness with normal range of motion in all extremities.

The patient received computed tomography (CT) scans of the chest, abdomen, and pelvis. The CT scan showed an enlarged prostate approximately 6.4 cm; otherwise, there were no other acute findings.

The initial hypothesis was a possible viral infection or malignancy, due to the enlarged prostate. However, a prostate-specific antigen (PSA) test was performed and returned negative. The patient tested negative for COVID-19, flu, and respiratory syncytial virus. Their urinary analysis was also unremarkable. On admission day, labs showed significant leukocytosis of 14.8 with a left shift (76% neutrophils).

On the second day, the patient received a brain magnetic resonance imaging (MRI) as well as a CT internal auditory canal (IAC). The MRI displayed a nonspecific right mastoid effusion and no acute intracranial abnormalities. The CT showed normal results except for chronic sclerosis in the right mastoid air cells. The patient tested negative for upper respiratory viral panel, HIV, syphilis, leukemia, and lymphoma panel, and their blood cultures showed no growth. The patient continued to experience fevers and began experiencing ataxia, confusion, and headaches. Hence, infectious diseases (ID) physician was consulted, and a cerebrospinal fluid (CSF) analysis using lumber puncture was recommended since the patient had abnormal white blood cell count. ID consulting physician also recommended a MRI of the cerebral spine with and without contrast to evaluate for spinal cord lesions. On this day, the patient was prescribed a 3-week regimen of ampicillin and 2 weeks of acyclovir.

On day three, the patient's condition remained unchanged. Further infectious workup for meningitis and encephalitis panel tested negative. CSF culture showed no growth, and CSF lymphoma leukemia panel was also negative.

On day four, the patient tested positive for an elevated ESR (73 mm/h) and CRP (22.70 mg/L). Consequently, both vasculitis and rheumatological workups were requested and results were as shown in Table 1. A repeat CXR showed no changes. The patient's family reported that the patient was experiencing mental fog and forgetfulness.

On days 5–7, the patient tested negative for *Histoplasma*, *Coccidioides*, *Blastomyces*, *Brucella*, *Coxiella*, *Bartonella*, Epstein–Barr virus, and tick panel. With the fevers persisting, an additional antibiotic, doxycycline, was added to the patient's regimen to be taken for 2 weeks. Also, the antiviral medication, acyclovir, was changed to valacyclovir. The oncology team was contacted to discuss a potential bone marrow biopsy to further work up unclear etiology of fevers. On day 7, the patient's platelet count was elevated at 425 K/μL.

On day 8, the bone marrow biopsy was completed. The patient now reported occipital and bitemporal headaches along with shoulder stiffness and visual disturbances which he stated he had been feeling intermittently since the onset of his fevers about 4–6 weeks ago. He did not share this valuable information earlier in the admission as he thought of the symptoms being unrelated to his fevers and were not persistent at the time. Vascular surgery was consulted for a temporal artery biopsy (TAB), and a computed tomography angiography (CTA) of the head, neck, and thoracic aorta was completed with pending results. The patient's platelet count continued to climb reaching 428 K/μL.

On day 9, a TAB was completed, and the patient was initiated on prednisone taper with 60 mg for 2 weeks, 50 mg for 2 weeks, 40 mg for 2 weeks, 30 mg for 1 week, 20 mg for 1 week, and 10 mg for 1 week; the patient had remained afebrile for the last 24 h and was discharged; close follow-up was advised with rheumatology and PCP. On this day, his platelet count peaked at 511 K/μL.

After the patient's discharge, the final test results became available. He tested negative for malignant epithelial cells. The marrow core biopsy returned negative for acute leukemia, definite lymphoma, and plasma cell neoplasm. The left TAB results were compatible with temporal arteritis. The microscopic examination showed a small muscular artery with the suggestion of inflammation in the vessel wall. Immunohistochemical analysis for CD3 and CD68 shows increased T-cells and histiocytes, respectively, throughout vessel wall and into adventitia. Hence, the above lab test confirmed the findings of GCA.

At 1 year follow up, the patient was following with rheumatology and his symptoms were well controlled while still on prednisone 10 mg daily. Upon lowering the dose any further he reported confusion and speech slurring and for which he had an extensive stroke workup completed, which was unremarkable. The patient subsequently continued low dose prednisone for symptom control and follow-up with rheumatology for further management.

## 3. Discussion

The primary risk factor for developing GCA is age, with the average age of presentation typically between 74 and 76 years, and an increasing incidence with age, peaking around 80 years [4]. Other risk factors include female sex, early menopause, polymyalgia rheumatica, smoking, low body mass index, or having a family history of GCA [4, 15, 16].

According to the 1990 American College of Rheumatology, some of diagnosing criteria for GCA were a patient being ≥ 50 years of age, an ESR ≥ 50 mm/h, a headache, and most importantly a positive TAB [17]. In 2011, Walvick and Walvick conducted a study and discovered that the odds of a positive biopsy for GCA were 1.5 times greater with an ESR over 47 mm/h, 5.3 times greater with a CRP > 2.45 mg/dL, and 4.2 times greater with platelets > 400,000/μL [18]. In 2019, Chan et al. conducted a similar study including 270 patients who were suspected to have GCA and received a TAB based on their elevated ESR (≥ 50 mm/h), CRP (20 mg/L), and/or platelet counts (300 × 10^9^/L). They found that the diagnostic accuracy of positive tests for ESR, CRP, and platelet counts for GCAwas very similar (*p* = 0.08). This study demonstrated that ESR, CRP, and platelets were equivalent as standalone diagnostic tests for GCA. However, notably, a combination of CRP and platelet test may provide the most diagnostic utility (*p* < 0.001) [19]. It was not until 2022 that the American College of Rheumatology's diagnosing criteria were updated to include a CRP of ≥ 10 mg/L as a diagnosing criterion of GCA [20]. Our patient tested positive for elevated ESR and CRP which both coincide with the 2022 diagnosing criteria. Also, according to both above studies, his elevated platelet count would be indicative of a possible GCA diagnosis.

**Figure 1:**
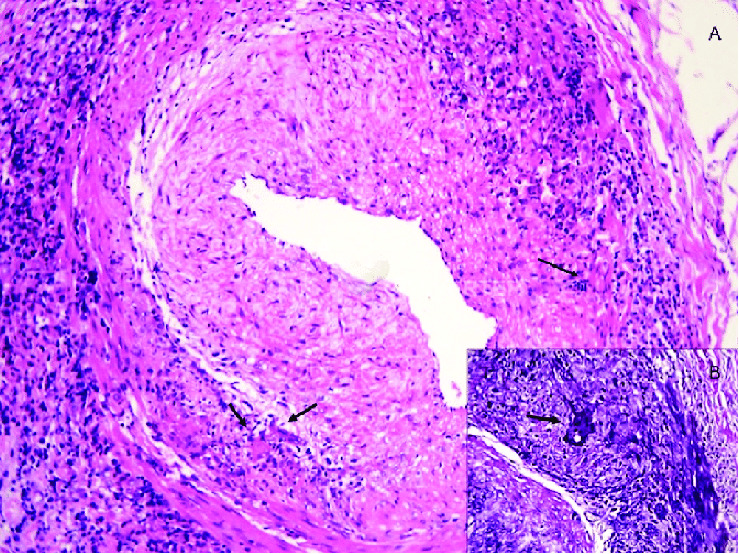
Pathology image from a patient with GCA who underwent temporal artery biopsy. (A) Anatomic pathology image (H/E 10x) of a temporal giant cell arteritis with inflammation of the vessel wall (active arteritis). Adventitious and subintimal fibrosis thickening. Inflammatory infiltrates in arterial wall. (B) Fragmentation and disruption of the internal elastic lamina with multinucleated giant cells (elastic stain) [14].

In the United States, TAB is thought of as being the gold standard for diagnosing GCA. As seen in Figure 1, a biopsy clearly shows inflammation of the vessel wall of the temporal artery and is diagnostic of GCA. However, there has been some debate regarding the accuracy of this test, with a report of up to 61% false negative results [21, 22]. To mitigate patients from undergoing this semi-invasive procedure, The European League Against Rheumatism has approved the use of imaging techniques such as ultrasound, MRI, CT, and positron emission tomography (PET) to detect the presence of extracranial involvement in GCA without cranial ischemic manifestations. Imaging techniques have been the key elements in redefining the diagnostic workup of GCA. In Europe, ultrasound of the temporal arteries is currently considered the main imaging tool for early diagnosis of GCA [23].

Our patient presented to the hospital stating they had experienced intermittent fevers for approximately 4 weeks. It is uncommon for an individual with GCA to present with fever as the sole symptom, so physicians did not suspect GCA as a diagnosis. Notably, this phenomenon was discussed in the case report by Poudel et al. which stated that presence of isolated persistent fever of unknown origin in patients over the age of 50 should suggest GCA even in the absence of characteristic clinical signs and symptoms [24]. To evaluate potential causes of recurring fever, an extensive workup including autoimmune, infectious, and hematologic testing in addition to imaging and lumbar puncture was obtained. All testing was unremarkable except for elevated ESR, CRP, and an elevated platelet count noted during the last few days of his hospital stay. It was not until the patient reported stiffness in their neck and shoulders as well as blurry vision that a TAB was suggested.

Diagnosing GCA remains difficult as patients typically present with nonspecific symptoms such as fever, headache, and joint pain. That coupled with the rarity of GCA in the general population contributes to physician anchoring bias resulting in a delay in diagnosis. Delay also occurs because patients may not be aware of the significance of symptoms, such as headache, jaw claudication, and associated joint pains in combination, and therefore do not seek help earlier in the disease course [25].

The average time for diagnosis from when symptoms present to when a patient is diagnosed with GCA is 9 weeks and it is even longer for those that do not experience cranial symptoms such as headache and temporal tenderness [5, 25]. One study found that 22% of those diagnosed with GCA lack cranial signs or symptoms [26]. Our patient received their diagnosis approximately 5 weeks after the onset of symptoms and would be considered an earlier diagnosis than average. As soon as the suspicion for GCA was established, treatment with steroids was initiated to achieve symptom control [13]. The patient reported resolution of fever soon after initiating treatment; lab results showed that his platelet count continued to trend down toward normal levels.

When the patient finally relayed what they thought were unrelated symptoms, the physicians were able to accurately diagnose and treat him. However, the patient's hospital stay was unnecessarily prolonged due to excessive testing before considering GCA. This could have been prevented with obtaining a thorough initial history and evaluation, which would have aided in the diagnosis process and avoided redundant tests. More education should be provided to the public about the risk factors of GCA for individuals over 50 years of age, especially since delayed diagnosis of this disease can be detrimental [27, 28].

## 4. Conclusion

• This case emphasizes the diagnostic challenges associated with GCA due to patients often presenting with nonspecific symptoms such as fever and headache leading to anchoring bias among treating physicians.
• In a patient over 50 years of age presenting with a fever of unknown origin, elevated ESR, CRP, and platelet count should lead to high index of suspicion for GCA.
• To help ensure a timely diagnosis, healthcare teams should obtain a detailed history addressing specific questions relating to symptoms such as headache, jaw claudication, shoulder stiffness, or blurry vision. Patients at times may feel these symptoms are unrelated to their condition and do not necessarily explain them during the initial physician encounter or the patient may be an unreliable historian and disregard these symptoms completely if they are not present at admission as noted in this case.
• An evidence-based approach is crucial in diagnosing GCA. This ensures thorough examination using insights from various specialties and the latest scientific evidence.

## Figures and Tables

**Table 1:** Patients lab results.

Test performed	Day results received	Patient's results	Normal range
Leukocytes	Day 1	14.8 × 10^9^/L	4.8–10.8 × 10^9^/L
Hemoglobin	Day 1	13.4 g/dL	12–16 g/dL
AST (aspartate transferase)	Day 1	204 U/L	0–48 U/L
ALT (alanine transaminase)	Day 1	239 U/L	13–61 U/L
ALP (alkaline phosphatase)	Day 1	165 U/L	45–136 U/L
INR (international normalized ratio)	Day 1	1.59	0.85–1.13
ESR (erythrocyte sedimentation rate)	Day 4	73 mm/h	0–20 mm/h
CRP (C-reactive protein)	Day 4	22.70 mg/L	0–0.6 mg/L
ANCA IFA titer	Day 4	< 1:20	Negative
Cyclic citrullinated peptide (CCP) antibody	Day 4	< 0.54	Negative (< 20 Units)
Rheumatoid factor (RF) numeric	Day 4	< 3.5 IU/mL	Negative (< 14 IU/mL)
ANA reflex to autoantibodies	Day 4	Negative	Negative
Platelet count	Day 7, 8, 9, 10, 43 days post discharge	425, 428, 511, 484, 226 K/μL	130–400 K/μL

## Data Availability

The data that support the findings of this study are available from the corresponding author upon reasonable request.
